# Supplementary material for: Surface Microbiome Profiling of Dental Elevators Using Third‐Generation Sequencing: Implications for Infection Control in Dental Practice
Source: Microbiologyopen. 2025 Dec 8;14(6):e70178. doi: 10.1002/mbo3.70178 (PMC12685768; doi:10.1002/mbo3.70178)
Supplement: Supplementary file 1 — customer_backup. [file MBO3-14-e70178-s001.zip › customer_backup/customer_backup/function_analysis/COG/allsample/K2species/allsample.reabundance_Class2Phylum.txt.pdf]

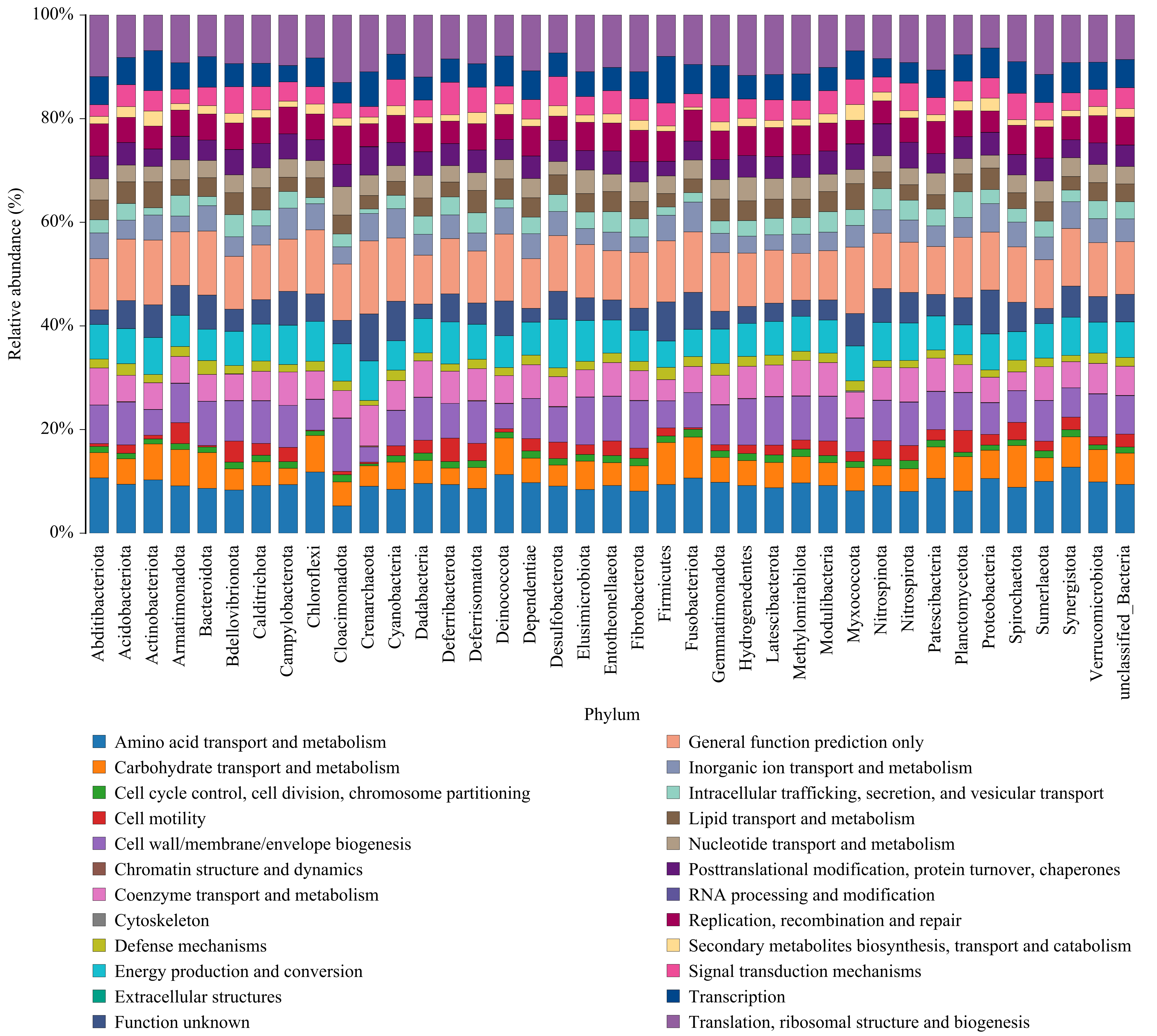
100%
80%
60%
40%
20%
0%
Relative abundance (%)
Abditibacteriota
Acidobacteriota
Actinobacteriota
Armatimonadota
Bacteroidota
Bdellovibrionota
Calditrichota
Campylobacterota
Chloroflexi
Cloacimonadota
Crenarchaeota
Cyanobacteria
Dadabacteria
Deferribacterota
Deferrisomatota
Deinococcota
Dependentiae
Desulfobacterota
Elusimicrobiota
Entotheonellaeota
Fibrobacterota
Firmicutes
Fusobacteriota
Gemmatimonadota
Hydrogenedentes
Latescibacterota
Methylomirabilota
Modulibacteria
Myxococcota
Nitrospinota
Nitrospirota
Patescibacteria
Planctomycetota
Proteobacteria
Spirochaetota
Sumerlaeota
Synergistota
Verrucomicrobiota
unclassified_Bacteria
Phylum
Amino acid transport and metabolism
Carbohydrate transport and metabolism
Cell cycle control, cell division, chromosome partitioning
Cell motility
Cell wall/membrane/envelope biogenesis
Chromatin structure and dynamics
Coenzyme transport and metabolism
Cytoskeleton
Defense mechanisms
Energy production and conversion
Extracellular structures
Function unknown
General function prediction only
Inorganic ion transport and metabolism
Intracellular trafficking, secretion, and vesicular transport
Lipid transport and metabolism
Nucleotide transport and metabolism
Posttranslational modification, protein turnover, chaperones
RNA processing and modification
Replication, recombination and repair
Secondary metabolites biosynthesis, transport and catabolism
Signal transduction mechanisms
Transcription
Translation, ribosomal structure and biogenesis
